# Elevated Beta-Human Chorionic Gonadotropin in a Non-pregnant Female With Altered Kidney Function

**DOI:** 10.7759/cureus.23747

**Published:** 2022-04-01

**Authors:** Iger Ostreni, Andreina Colatosti, Eric J Basile, Omar Rafa

**Affiliations:** 1 Medicine, Touro College of Osteopathic Medicine, New York, USA; 2 Obstetrics and Gynecology, Brooklyn Hospital, Brooklyn, USA; 3 Internal Medicine, University of Florida College of Medicine, New York, USA; 4 Internal Medicine, University at Buffalo, Buffalo, USA

**Keywords:** gynecology and obstetrics, beta-hcg, acute kidney injury, acute kidney failure, adverse pregnancy outcome, ob-gyn, general obstetrics

## Abstract

Elevated beta-human chorionic gonadotropin (beta-hCG) levels in postmenopausal women is a finding known in the literature; however, it still commonly leads to unnecessary and extensive diagnostic workup. We present the case of a 48-year-old African-American postmenopausal female with acute kidney injury on chronic kidney disease (CKD) stage 5 and an incidental finding of elevated serum beta-hCG. Abdominal and transvaginal ultrasound showed no evidence of intrauterine or ectopic pregnancy or gestational trophoblastic disease. Menopausal status was confirmed with follicle-stimulating hormone (FSH) measurement, and following the improvement of renal status, beta-hCG levels were normalized to expected values for the patient’s age group. The etiology of elevated beta-hCG was suspected to be from the pituitary as previous literature has shown decreasing beta-hCG levels in postmenopausal women following the administration of gonadotropin-releasing hormone (GnRH) antagonist.

## Introduction

Human chorionic gonadotropin (hCG) is a heterodimeric hormone consisting of an alpha (α) and a beta (β) subunit. It is produced in small amounts by the pituitary and other organs, including the testis, liver, and colon, and in much larger amounts by placental trophoblast and malignancies such as hydatidiform mole, choriocarcinoma, and germ cell tumors [1]. Therefore, hCG is a useful clinical marker for detecting and monitoring various physiologic and pathologic conditions [2]. The most common causes of elevated serum beta-hCG are pregnancy and trophoblastic tumors. The hCG molecules are differently metabolized by the liver, ovary, and kidney, but the majority of hCG forms are excreted in the urine [3]. After ruling out pregnancy and malignancy as the cause of elevated serum beta-hCG, a positive result may also be explained by a less common cause, as described in this patient’s case.

## Case presentation

**Figure 1:**
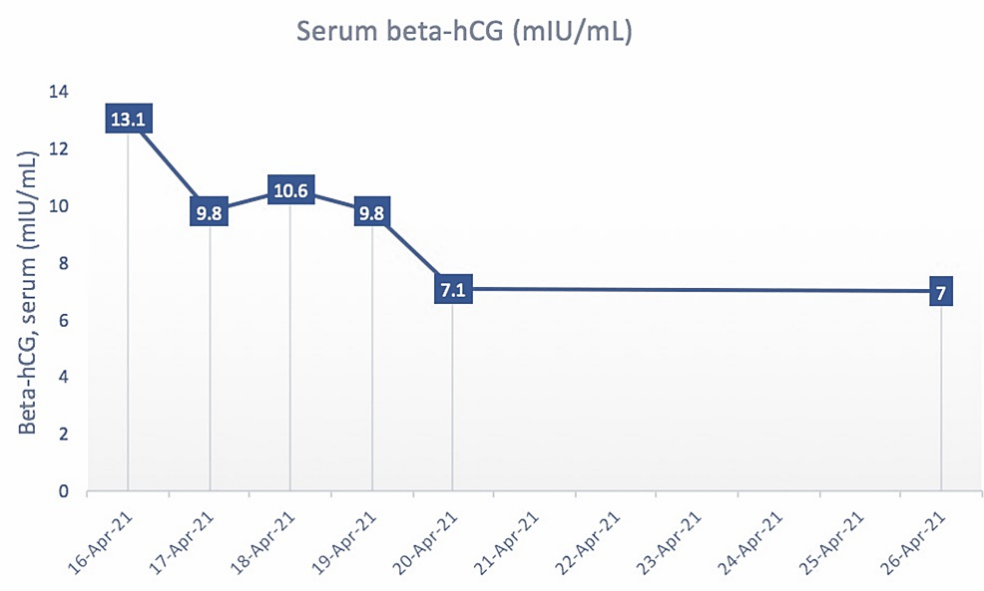
Graph depicting serum beta-hCG levels of the patient over the course of her hospital admission in mIU/mL.

**Figure 2:**
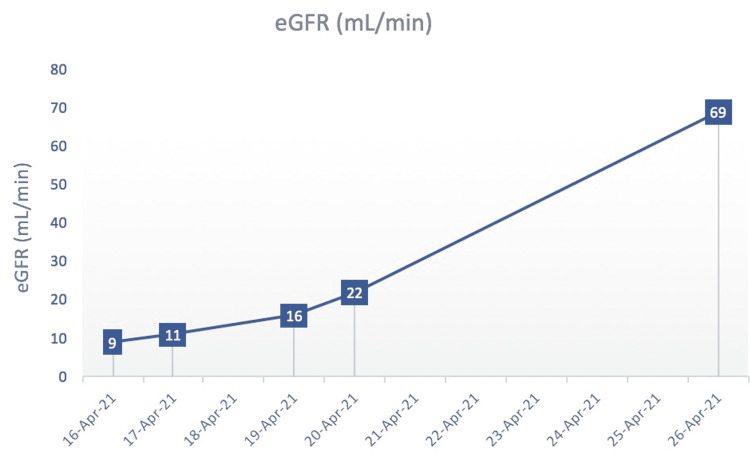
Graph depicting the estimated glomerular filtration rate (eGFR) of the patient over the course of her hospital admission in mL/minute.

The patient is a 48-year-old African-American postmenopausal female with a past medical history of chronic kidney disease (CKD) stage 5 on hemodialysis and diabetes mellitus type 2, who presented from a skilled nursing facility to the emergency department with intractable vomiting, nausea, and abdominal pain for one week’s duration. She was found to have acute kidney injury on CKD and electrolyte abnormalities due to dehydration. As part of her initial workup, serum beta-hCG was ordered to rule out pregnancy prior to computed tomography (CT). Incidentally, beta-hCG was found to be elevated with a level of 13.1 mIU/mL (Figure 1). The patient reported that her last menstrual period was three years ago, and her last sexual encounter was six months prior to her hospital presentation. Transabdominal ultrasound revealed an anteverted uterus with normal echogenicity and morphology and unremarkable left and right ovaries. The endometrial echo complex measured 0.5 cm, which was normal in thickness. CT of the abdomen and pelvis with oral contrast revealed no evidence of acute intra-abdominal or pelvic pathology. Transvaginal ultrasound was ordered and showed no evidence of intrauterine gestation. Pertinent laboratory studies showed an estimated glomerular filtration rate (eGFR) of 9 mL/minute and creatinine of 5.7 mg/dL (normal: 0.7-1.5 mg/dL) on admission. The patient was started on intravenous hydration with significant improvement in kidney function as measured by eGFR. During her 10-day hospital course, her eGFR improved to 69 mL/minute, and her levels of beta-hCG decreased to 7 mIU/mL (Figure 2).

## Discussion

Beta-hCG is a well-established marker in the detection and viability of early pregnancy. The hormone is produced mainly by syncytiotrophoblastic cells during pregnancy, and smaller amounts are also produced physiologically by the pituitary, testis, liver, and colon [4]. Malignancies known to be associated with elevated levels of beta-hCG include ovarian germ cell tumors, gestational trophoblastic neoplasia, and non-trophoblastic tumors (lung, oral/facial, breast, cervical, ovarian, endometrial, vulvar/vaginal, colorectal, prostate, pancreas, renal, and neuroendocrine) [5]. Values of beta-hCG in the indeterminate (5-25 IU/L) or positive range (>25 IU/L) prompt additional workup to rule out pregnancy or malignancies [6]. Additionally, elevated serum beta-hCG has also been a reported finding in postmenopausal females, with 6%-8% having an increased value; however; it is not a well-known trend (Table 1) [7].

**Table 1:** Expected serum hCG levels in females corresponding to different ages and conditions. Basham et al. [7]

Category	hCG levels (IU/L)
Premenopausal (aged 18–40 years), non-pregnant	<5
Perimenopausal (aged 41–55 years), non-pregnant	<8
Postmenopausal (aged >55 years)	<14
Early normal pregnancy	>50
Pregnancy, second–third trimester	27,300–233,000
Gestational trophoblastic neoplasia	>100,000

In otherwise healthy postmenopausal females, the source of increased beta-hCG has been attributed to pituitary release. Daiter et al. showed that persistently elevated levels of beta-hCG in a postmenopausal female increased by 50% following the administration of a synthetic gonadotropin-releasing hormone (GnRH) agonist [8]. Then, the administration of a GnRH antagonist led to a subsequent decrease in serum beta-hCG. The conclusion was that the pituitary was the source of elevated beta-hCG concentration as the pituitary is exposed to the highest concentration of GnRH endogenously.

Furthermore, the pituitary release of beta-hCG is correlated with increased levels of luteinizing hormone (LH) and follicle-stimulating hormone (FSH) in postmenopausal women. LH and FSH levels should be obtained to determine the source of the beta-hCG. If LH and FSH levels are close to or above the reference limit for menopause, it is likely that the source is from the pituitary. Additional confirmatory workup includes a trial with hormone replacement therapy. Estradiol supplementation can be given for a period of at least three weeks [9] to lower the interference from the pituitary and determine if the source is an ectopic secretion [10].

Additional sources of elevated serum beta-hCG include a variety of causes, such as heterophile antibodies [11], rheumatoid factors, IgA deficiency [12], chronic renal failure or end-stage renal disease (ESRD) on hemodialysis [11], red blood cell or plasma transfusions with hCG in it [13], and exogenous hCG preparations for weight loss and doping [14]. Heterophile antibodies can be present in the serum and interfere with the antibodies used in hCG tests. Furthermore, 1/3,300 women may have a high enough concentration of these antibodies interfering with the radioimmunoassay [11]. A urine test can serve as a confirmatory beta-hCG measurement in these cases.

In the setting of renal failure, the presence of an elevated beta-hCG in end-stage renal disease (ESRD) without pregnancy may be caused by decreased metabolic clearance due to poor renal filtration and elimination [15,16]. Algorithms have been generated for the evaluation of beta-hCG levels in patients with ESRD with suspicion of pregnancy [3,17].

However, once pregnancy has been ruled out, no clear consensus exists on how to evaluate the source of beta-hCG in these patients, particularly if they have a superimposed acute kidney injury. Other causes of elevated beta-hCG can be evaluated on a case-by-case basis by obtaining a good medical history to determine if further workup is necessary.

## Conclusions

This case highlights the importance of proper patient management following elevated levels of beta-hCG in postmenopausal women. Increased values were placed in the context of the patient’s age, menopausal status, and medical history. Increased FSH values confirmed the patient’s menopausal status and the pituitary as the likely source. Resolution of the kidney injury also resulted in normal beta-hCG levels according to her age group.

Of note, it is important to state that if there were no signs of values trending down, the next step is to confirm if the source of beta-hCG is originating from the pituitary by administering an estrogen suppression test. If unsuppressed, then it may be acceptable to continue workup for malignancy. Understanding the potential etiologies for elevated beta-hCG aids in broadening the differential and helps contextualize the patient’s clinical picture.
